# Synthesis and Evaluation of the Anxiolytic Activity of Some Phthalimide Derivatives in Mice Model of Anxiety

**Published:** 2012

**Authors:** Farshid Hassanzadeh, Mohammad Rabbani, Ghadam Ali Khodarahmi, Mehrnoosh Moosavi

**Affiliations:** a *Department of Medicinal Chemistry, Isfahan University of Medical Sciences, Isfahan, Iran.*; b *Department of Pharmacology, Isfahan Pharmaceutical Sciences Research Center, Iran.*; c *Isfahan Pharmaceutical Sciences Research Center, Faculty of Pharmacy, Isfahan University of Medical Sciences, Isfahan, Iran.*

**Keywords:** Phthalimide, 3-Nitrophthalimide, Elevated plus-maze, Anxiety

## Abstract

The aim of the present study was to synthesis a series of phthalimides based on our previous works and examine their anxiolytic properties. Using a three steps process, phthalimides were prepared from the corresponding di-methyl phthalate derivatives. Phthalic anhydride was nitrated to produce 3-nitrophthalic acid. Ring closer of either 3-nitrophthalic acid or di-methyl phthalate with urea were carried out in reflux condition. Final compounds were prepared by base catalyzed condensation of 4-methylbenzoyl chloride, benzoyl chloride and benzyl chloride with the resulting imides. From the tested compounds, only *N-*benzoyl 3-nitro-phthalimide was shown to produce anxiolytic activity by increasing the number of entries and time spent in open arms at 10 mg/kg.

## Introduction

Benzodiazepines are widely used as anxiolytic agents. A flat aromatic structure containing an electron withdrawing group at proper position (C_7_ on ring A) is necessary for benzodiazepine’s activities (1). Barbiturates which are used as sedative agents have flat structures containing imide functional groups (2). N-Benzoyl-phthalimide resembles both classic benzodiazepines and barbituric acids structures “two major families of sedatives and anxiolytics”. N-Benzoyl-phthalimide consists of a tricyclic hydrophobic structure comparable to that of benzodiazepines and possesses a conjugated ureid functional group as can be found in barbiturates (3). N-Benzoyl phethalimide was synthesized previously in our department and tested positive for anxiolytic activity at 0.5 mg/kg dose in mice. In this study we aimed to synthesize and test some new derivatives of N-benzoyl phthalimide. 

To examine the minor structural tuning on biological activity, introduction of an electron withdrawing moiety (NO_2_) on ring A, and or an electron donating group (CH_3_) on ring C and enhancing the flexibility of the structure by replacing the C=O bridge with methylen group are aimed. 

## Experimental

*Chemistry*

All reagents and solvents used were of general purpose grade. Melting points were determined on an electrothermal 9200 apparatus and are uncorrected. Infra-red spectra were obtained as solid via a diffuse reflectance accessory using KBr matrix, using a Perkin Elmer 1420 series. ^1^H-NMR spectra were recorded on a Bruker FT-80 spectrometer as dilute solutions in CDCl_3_ or DMSO-d_6_ with tetramethysilane as internal standard. Mass spectra were recorded on a fisons trio 1000 GC-Mass. 

**Figure 1:**
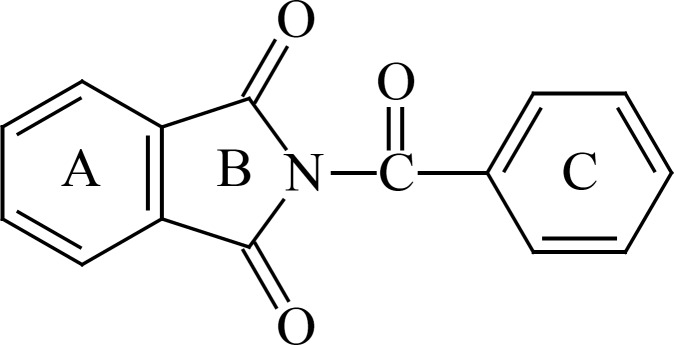
*N*-Benzoyl-phthalimide

*Phthalimide*

Dry urea (15 g, 0.25 mol) was added stepwise to a mixture of dimethyl phthalate (19.4 g, 0.1 mol) in 50 mL sodium methoxide solution and was stirred at reflux condition for 6 h. The resulting white suspension was concentrated under reduced pressure at 50 °C. The residue was dispersed in 100 mL of ice water mixture and neutralized with diluted HCl and filtered. Precipitates were re-crystallized from ethanol to give the imide, as white crystals (9.19 g, 46.3 %), m.p. 228–230 °C (Lit., [6]), 233-234 °C) ;* v *_max_ 3200 (N-H), 1720 (C=O), 1600 (C=C, Ar) cm^-1^; δ_H_ (80 MHz ; CDCl_3_), 11.2 - 10.8 (1H, br s, NH), 7.8 (4H, s, ph-H).

*N-(4’-Methylbenzoyl) -phthalimide*

A mixture of phthalimide (2 g, 13.6 mmol) and 4-methylbenzoyl chloride (2.1 g, 13.6 mmol) was refluxed in dry acetone (50 mL) containing potassium carbonate (10 g, 0.1 mol) for 4 h to produce N-(4’-methylbenzoyl)-phthalimide as white crystals. Yield 40%, m.p. 204 °C; [Found: M, 265. C_16_H_11_NO_3 _requires M, 265]; *v*_max_ 1780 - 1680 (C=O ), 1590 (C=C,Ar); δ_H_ (80 MHz, CDCl_3_), 8.1–7.7 (6H, m, COC=CH-CH=CH-CH=CCO, CH-CC=O-CH ), 7.4–7.2 (2H, d, J=8 Hz, CH-CCH_3_-CH), 2.5 (S, 3H, CH_3_).

**Figure 2:**
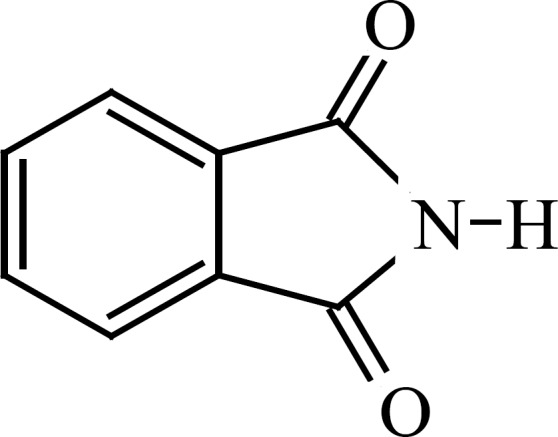
Phthalimide

*3-Nitro Phthalic acid*

To a preheated (to 80 °C) mixture of phthalic anhydride (100 g, 0.675 mol) and concentrated sulfuric acid (100 mL), a mixture of fuming nitric acid (42 mL) and concentrated sulfuric acid (30 mL) was added drop-wise. The mixture was added to sufficient amount of crashed ice to obtain the 3-nitro compound as a yellow solid. The yield was 30%, m.p. 212°C (Lit., [4,5]), 208-210 °C); *v*_max_ 3500-2500 (OH), 1720 - 1680 (C=O ), 1600 (C=C,Ar), 1550, 1350 (NO_2_); δ_H_ (80 MHz, DMSO-d6), 8.4 – 8.3 (1H, d, J = 8 Hz CNO_2_-CH), 8.2 – 8.1 (1H, d, J = 8 Hz, CO-C-CH-CH), 7.9–7.7 (1H, t, J = 8 Hz, CO-C-CH-CH).

*3-Nitro-Phthalimide*

A mixture of 3-nitro phthalic acid (21.1 g, 0.1 mol) and urea (6 g, 0.1 mol) was refluxed in ethylene glycol mono methyl ether (40 mL) for 12 h. The resulting mixture was transferred to a beaker containing crashed ice to deposit the 3-nitro derivative as a yellow powder which was recrystalized in ethyl acetate. Yield 67%. m.p. 223 °C; *v*_max_ 3150 (NH), 1780 - 1680 (C=O ), 1600 (C=C,Ar), 1550–1350 (NO_2_); δ_H_ (80 MHz, DMSO-d6), 11.8–11.6 (1H, br, NH), 8.2–7.9 (3H, m, Ph-H).

*N-Be* *nzoyl-3-Nitro-Phthalimide*

A mixture of -3-nitro-phthalimide (1.92 g, 0.01 mol) and benzoyl chloride (1.40 g, 0.01 mol) was refluxed in dry acetone (50 mL) containing potassium carbonate (5 g) for 6 h to produce N-benzoyl 3- nitro- phthalimide as yellow crystal. Yield 50%. m.p. 199 °C; [Found: M, 296. C_15_H_10_N_2_O_4_ requires M, 296];* v*_max_ 3060 (CH), 1800 -1670 (C=O ), 1600 (C=C, Ar), 1530–1350 (NO_2_); δ_H_ (80 MHz, CDCl_3_), 8.5–8 (5H, m, CNO_2_=CH-CH=CH, CH-CCO=CH), 7.8–7.5 (3H, m, CH-CCO=CH-CH=CH-CH).

*N-(4’-Methylbenzoyl)-3-Nitro-phthalimide*

A mixture of 3-nitro-phthalimide (1.92 g, 0.01 mol) and 4-methylbenzoyl chloride (1.54 g, 0.01 mol) was refluxed in dry acetone (50 mL) containing potassium carbonate (10 g, 0.1 mol) for 4 h to produce N-(4-methylbenzoyl)-3-nitro-phthalimide as white crystals. Yield 48%. m.p. 150 °C; [Found: M, 310. C_15_H_11_BrNO_2 _requires M, 310]; *v*_max_ 3020 (CH), 1770-1690 (C=O), 1600 (C=C,Ar), 1530–1370 (NO_2_); δ_H_ (80 MHz, DMSO-d6), 8.1–7.6 (5H, m, CNO2=CH-CH=CH, CH-CCO=CH), 7.6 – 7.3 (2H, d, J=8Hz, CH-CCH_3_=CH), 2.4 (3H, s, CH_3_).

**Figure 3:**
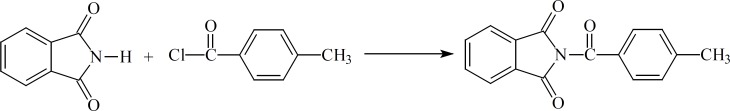
*N*-(4'-Methylbenzoyl) -phthalimide

**Figure 4:**
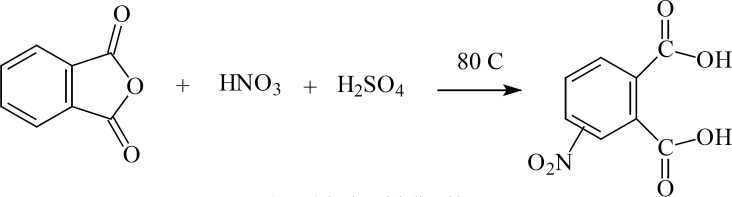
3-Nitro Phthalic acid

*N-Benzyl -3-Nitro-Phthalimide*

A mixture of 3-nitro-phthalimide (0.75 g, 0.003 mol) and benzyl chloride (0.4 g, 0.003 mol) was refluxed in dry acetone (50 mL) containing potassium carbonate (5 g, 0.05 mol) for 3 h to produce N-benzyl 3-nitro-phthalimide as yellow crystals. Yield 13.3%. m.p. 100 °C; [Found: M, 282. C_15_H_10_N_2_O_4 _requires M, 282]; *v*_max_3040 (C-H, Ar), 2925 (C-H), 1780 - 1700 (C=O), 1600 (C=C, Ar), 1530 – 1370 (NO_2_); δ_H_ (80 MHz, CDCl_3_), 8.2 – 7.6 (3H, m, PhNO_2_-H), 7.5–7.1 (5H, m, ph-H), 4.9 (2H, s, CH_2_).

*Pharmacology*

*Animals*

Male NMRI mice (Pasture, Tehran) weighing 25-30 g were housed in a cage with controlled room temperature at 22-25°C. Food and water were available *ad libitum*. Tests were performed only after the mice had been acclimatized to the above environment for at least 7 days. All experiments were carried out between 09:00 and 13:00 h. Each mouse received a single intraperitoneal (IP) injection of drug or vehicle and was tested once in the EPM. 

*Elevated plus-maze*

The EPM test is described in details elsewhere (6-9). Briefly, the apparatus comprised of two open arms (35 5 cm) and two closed arms (30 5 15 cm) that extended from a common central platform (5 5 cm). The floor and the walls of each arm were wooden and painted black. The entire maze was elevated to a height of 50 cm above floor level as validated and described by Lister (Lister, 1987). Testing was conducted in a quiet room that was illuminated only by a dim light. Mice were given a single ip dose of various test compounds or diazepam (Sobhan Pharmaceutical Co. Iran) 30 min before their placement on the EPM. To begin a test session, mice were placed on the open arm facing the center of the maze. An entry into an arm was defined as the animal placing all four paws over the line marking that area. The number of entries and the time spent in the open and closed arms were recorded during a 5 min test period. The percentage of open arm entries (100 open/total entries) was calculated for each animal. Between each trial, the maze was wiped clean with a damp sponge and dried with paper towels.

**Figure 5:**
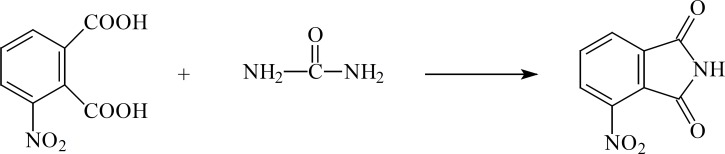
3-Nitro-Phthalimide

**Figure 6:**
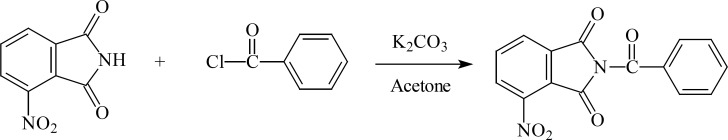
*N*-Benzoyl 3-Nitro-Phthalimide

*Statistics*

Statistical analysis was performed using one-way analysis of variance (ANOVA) with post hoc Tukey test. P < 0.05 was considered significant. All data are expressed as mean ± standard error of mean (SEM).

**Figure 7:**
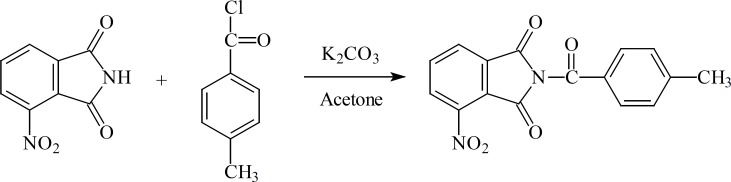
*N*-(4'-Methylbenzoyl)-3-Nitro-phthalimide

## Results

*Elevated plus-maze results*

Diazepam at the dose of 1.5 mg/kg significantly increased both the time and the number of entry in to the open arms (Figures 9 and10). Various doses (5, 10 and 50 mg/kg) of phthalimide derivatives namely, *N*-benzoyl 3-nitro-phthalimide, *N*-benzyl 3-nitro-phthalimide and *N*-(4›-methylbenzoyl)-3-nitro-phthalimide were tested for anxiolytic properties using EPM model. The only compound that significantly increased the time and entries into the open arm was *N*-benzoyl 3-nitro-phthalimide at 10 mg/kg (Figures 9 and 10 p < 0.01). The anxiolytic properties of the *N*-benzyl 3-nitro-phthalimide, studied in a separate set of experiments showed to be ineffective in changing the EPM parameters (data not presented).

## Discussion

The aim of the present study was evaluation of the anxiolytic actions of some phthalimide derivatives in comparison to that of diazepam. As expected, diazepam produced significant increases in open arm time and in number of entries into the open arms. These data are consistent with the results of numerous previous studies, which have shown that diazepam and other benzodiazepines produce robust anxiolytic effects in a variety of anxiolytic screening procedures. Among the derivatives of phtalimide that were tested in this study only *N*-benzoyl 3-nitro-phthalimide produced anxiolytic action, the effect that was seen with an increase in time spent on the open arm and the number of entries. This effect of the phtalimide derivative was produced at a dose of 10 mg/kg which is higher than that of diazepam (2.5 mg/kg).

**Figure 8:**
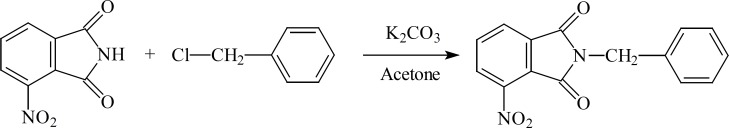
*N*-Benzyl 3-Nitro-Phthalimide

*N*-Benzoyl-3-nitro-phthalimide showed a lower activity compare to that of diazepam and *N*-benzoyl phthalimide that was tested in our previous study and showed to be effective at 0.5 mg/kg (3). An electron withdrawing group (Cl, NO_2_) on C_7_ of benzodiazepines is essential for sedative and anxiolytic activities of classic benzodiazepine agonists. Substitution of Cl or NO_2_ on other positions of the aromatic ring (6, 8, 9) of benzodiazepines dramatically reduce activity (1). Alpidem (an imidazopyridazine partial agonist) which is a selective anxiolytic (one-eighths as potent as diazepam), without sedative and muscle relaxant effects of benzodiazepines has a flat structure with an electron withdrawing group (Cl) at meta position of ring A (10). Reduction in activity of *N*-benzoyl, 3-nitro-phthalimide might be due to the improper accommodation of electron withdrawing group (NO_2_) in the benzodiazepine active site. Substitution of an electron donating group (CH_3_) on the C ring of the parent compound in *N*-(4’-methylbenzoyl)-3-nitro-phthalimide and *N*-(4’-methylbenzoyl)-phthalimide was also in favor for anxiolytic activity, this also has been seen in benzodiazepine series. Substitution at the 4’-(para)-position of the phenyl ring of benzodiazepines is unfavorable for agonist activity; however, 2’-(ortho)-substituents are not detrimental to agonist activity.

**Figure 9:**
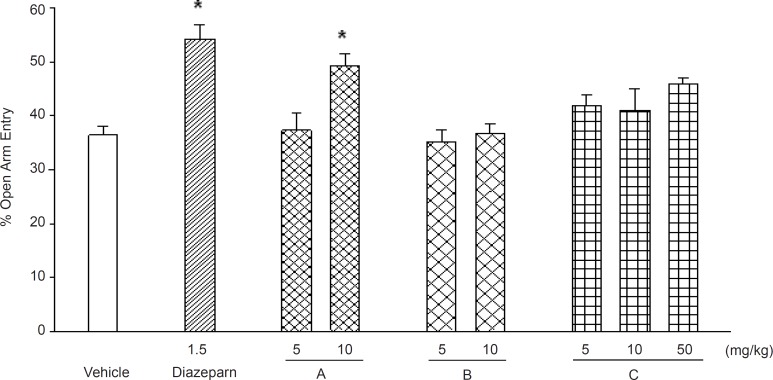
Effects of diazepam and the test compounds "*N*-benzoyl 3-nitro-phthalimide (A), *N*-(4'-methylbenzoyl)-3-nitro-phthalimide (B) and *N*-(4'-methylbenzoyl) -phthalimide (C), on the open arm entries of the EPM during a 5 min test in mice. Data are presented as mean values (± SEM) from group of 6 mice p <.0.05 compared with vehicle-treated control

**Figure 10:**
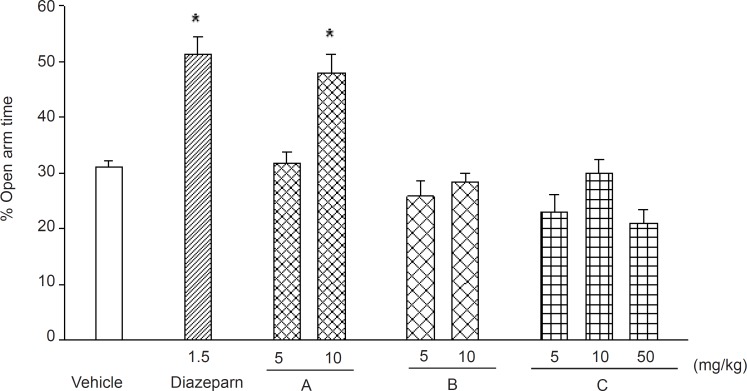
Effects of diazepam and the test compounds: "*N*-benzoyl 3-nitro-phthalimide (A), *N*-(4'-methylbenzoyl)-3-nitro-phthalimide (B) and *N*-(4'-methylbenzoyl)-phthalimide (C), on the time spent in the open arms, of the EPM during a 5 min test in mice. Data are presented as mean values (± SEM) from group of 6 mice. *p < 0.05 compared with vehicle-treated control

**Figure 11:**
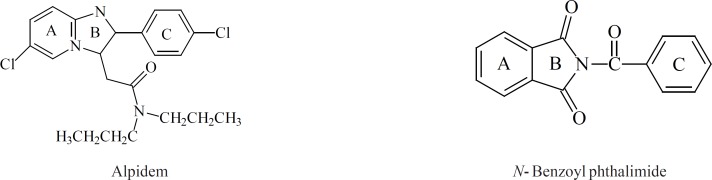
Alpidem and *n*- Benzoyl phathalimide.

*N*-Benzyl 3-nitro-phthalimide distorted from planarity due to the change of C=O group of *N*-benzoyl phthalimide to CH_2_ group. This distortion probably prevents the accommodation of the compound with its receptor and makes the compound ineffective as an anxiolytic agent. In benzodiazepines, the phenyl ring is attached directly to the ring B and its relationship to the ring A planarity may be important for agonist activity. All GABA_A_ partial agonists including imidazopyridines (alpidem and zolpidem) also have flat structures.
